# Fragmented ownership and multilevel barriers to breast MRI screening among high-risk women: A qualitative study informing development of clinical decision support

**DOI:** 10.21203/rs.3.rs-10591503/v1

**Published:** 2026-09-20

**Authors:** Marlee Pincus, Cynthia Law, Vicky Ro, Christina Hijiya, Rita Kukafka, Katherine D Crew, Alissa Michel

**Affiliations:** Columbia University Irving Medical Center; Herbert Irving Comprehensive Cancer Center; Herbert Irving Comprehensive Cancer Center; Columbia University Irving Medical Center; Herbert Irving Comprehensive Cancer Center; Herbert Irving Comprehensive Cancer Center; Herbert Irving Comprehensive Cancer Center

**Keywords:** Breast MRI screening, breast cancer risk assessment, shared decision-making, barriers to enhanced cancer screening, care coordination

## Abstract

**Background:**

Women at high risk for breast cancer include those with pathogenic variants in hereditary breast cancer predisposition genes, high-risk benign breast lesions, or a lifetime risk over 20% based on validated models. Supplemental breast MRI screening improves early detection and reduces interval cancers in high-risk women. Despite recommendations from professional organizations for breast MRI screening among high-risk women, uptake remains low. To better understand breast MRI utilization, we conducted a qualitative study exploring healthcare provider and patient perspectives to inform development of a screening module within our patient-facing web-based decision aid, *RealRisks*.

**Methods:**

We conducted semi-structured interviews with 20 women at high risk for breast cancer and 17 healthcare providers at an urban academic medical center in New York City. All interviews were transcribed verbatim and coded using Dedoose software. Transcripts were analyzed thematically to identify patterns in clinician communication strategies, MRI screening practices, as well as barriers and facilitators shaping implementation.

**Results:**

All enrolled providers were female, including six primary care providers (PCPs), five gynecologists, four breast radiologists, and two cancer genetic counselors. Among enrolled patients, mean age was 51.8 years (SD, 14.8) and 40% were Hispanic, 40% non-Hispanic White, 10% non-Hispanic Black, and 10% other. More than half of providers (59%) had ordered a breast MRI for high-risk patients, and 55% of patients had previously undergone MRI screening. The three themes that emerged included: 1) responsibility in initiating MRI screening was fragmented across specialties; 2) patients and providers encountered barriers at every step of the screening process, including knowledge gaps and uncertainty about insurance coverage; 3) both groups identified a need for clearer, consolidated guidelines and accessible resources to support informed decision-making.

**Conclusion:**

Our findings highlight the need to address structural and communication barriers to improve MRI screening uptake among high-risk women and their providers. Integrating electronic medical record (EMR)-based risk assessment and patient educational materials into clinical workflows may support informed, shared decision-making. Future work will involve design and usability testing of the *RealRisks* screening module, which will be available in English and Spanish and tailored to patients with varying health literacy and educational backgrounds.

## Background

Breast cancer is the most common malignancy among women worldwide and a leading cause of cancer-related mortality [1]. Women at elevated risk represent a heterogenous group and include those with pathogenic variants in hereditary breast and ovarian cancer (HBOC) genes, high-risk benign breast lesions, or a lifetime risk > 20% based on established risk assessment models [2].

Mammography has particular limitations in women with extremely dense breasts, who face a 4–5 fold increased risk of breast cancer and in whom dense tissue can obscure lesions, reducing mammographic sensitivity [3]. Randomized controlled trials support the addition of breast magnetic resonance imaging (MRI) to mammography screening in high-risk populations [4]. The DENSE trial demonstrated an approximately 50% reduction in interval breast cancers among women with extremely dense breasts, while the FaMRIsc trial showed improved detection and earlier-stage diagnoses in women with familial risk [4, 5]. The WISDOM trial evaluated risk-stratified breast cancer screening, including supplemental breast MRI among women at elevated risk and found that personalized, risk-based screening approaches are feasible and non-inferior to broad, one-size-fits-all strategies with annual mammography [6]. These findings taken together provide a conceptual foundation for tailoring screening and, specifically, supplemental MRI recommendations.

Guidelines from the National Comprehensive Cancer Network (NCCN) and the American College of Radiology (ACR) recommend supplemental MRI screening for high-risk women [7-9]. However, uptake remains low, ranging from 23–32% [10]. Barriers include limited use of formal breast cancer risk assessment, insufficient provider and patient knowledge about supplemental screening, and challenges accessing care [11]. Prior studies have shown that primary care providers (PCPs) frequently rely on family history–based clinical judgment rather than validated risk prediction models, with only 12% recommending MRI screening for eligible high-risk women [12]. In contrast, MRI completion rates are substantially higher in specialized high-risk clinics (88% vs 33%), highlighting the role of provider expertise in facilitating screening uptake [10]. Significant disparities exist in access to MRI screening, with non-Hispanic White women more likely to undergo screening than women from other racial and ethnic groups, and individuals with lower educational attainment less likely to receive supplemental screening [13].

To address these gaps in breast cancer risk assessment and management, our team previously developed two complementary web-based decision support tools: *RealRisks*, a patient-facing web-based decision aid (DA) that delivers personalized breast cancer risk estimates and education, and *BNAV (*Breast cancer risk NAVigation tool), a provider-facing platform with modules on risk assessment, screening and risk-reducing strategies [14]. Both tools have been shown to improve breast cancer risk perception, knowledge, and informed decision-making in diverse populations [15-17]. Building on this work, we now aim to refine the existing tools by incorporating content focused on breast cancer screening, with the goal of incorporating information for risk-based approaches for women with varying breast cancer risk profiles. The objective of this qualitative study was to better understand barriers to breast MRI utilization through semi-structured interviews exploring provider and patient perspectives to inform development of a screening module within our web-based DA, *RealRisks*.

## Methods

### Participant Recruitment

We conducted semi-structured interviews with two groups: (1) healthcare professionals (PCPs, gynecologists, breast radiologists, and cancer genetic counselors) caring for women at elevated cancer risk, and (2) women at high risk for breast cancer, including those with pathogenic variants in hereditary breast cancer predisposition genes, high-risk benign breast lesions, or a lifetime risk over 20% based upon the Gail or Tyrer-Cuzick models [18]. Eligible patients were English- or Spanish-speaking women aged ≥ 21 years without a personal history of breast cancer. Participants were prospectively recruited from June to November 2025 via (1) flyers in outpatient clinics at Columbia University Irving Medical Center (CUIMC) and (2) review of breast clinic schedules. The study was approved by the Institutional Review Board at CUIMC.

### Interviews

Two semi-structured interview guides (for providers and patients) were developed by the study team and informed by literature on family history screening, breast cancer risk assessment, and genetic testing. The interview guides were informed by the Theoretical Domains Framework which identifies 14 domains influencing health professional behavior including knowledge, skills, beliefs about consequences and context. The framework supported systematic identification of modifiable barriers and facilitators across provider and patient groups [19]. The guides covered domains including current practices and patterns as well as decision-making needs. Interviews were designed to elicit open-ended responses and lasted approximately 30–60 minutes.

### Data Collection

Participants completed a pre-interview online survey to collect demographic information. Provider surveys captured age, sex, race/ethnicity, medical specialty, years in practice, use of breast cancer risk assessment tools (yes/no), and whether they ordered screening breast MRI (yes/no). Patient surveys captured age, race/ethnicity, whether breast cancer risk had been discussed with them (yes/no/unsure), and whether they had undergone screening breast MRI (yes/no/unsure). Interviews were conducted virtually by multiple research team members (CL, AM, VR, CH). With participant consent, interviews were audio-recorded and initially transcribed using Microsoft Teams software. Final transcripts were reviewed for accuracy by study team members (CL, AM, VR, CH). Sample size was guided by the principle of information power [20]. Given the study’s narrow aim, the specificity of the sample (providers directly involved in breast cancer screening and patients meeting high-risk criteria), and the quality of dialogue achieved in interviews, we determined that 20 patients and 17 provider interviews provided sufficient information power to develop robust themes.

### Data Analysis

Data was managed using Dedoose software (Version 10.0.59; SocioCultural Research Consultants, LLC, 2023). Coding was conducted as an interactive, reflexive process, consistent with Braun and Clarke’s six-phase framework [21]. Analytic rigor was supported through independent coding of an initial subset of transcripts (providers: CL, AM; patients: VR, CH), regular team discussions to interrogate assumptions and followed by discussion to develop a preliminary codebook. The codebook was iteratively refined as coding progressed. Themes were developed through discussion and finalized collaboratively.

## Results

A total of 20 patients at high risk for breast cancer and 17 health care providers participated in the semi-structured interviews (Tables 1 and 2). The 17 participating providers included 6 primary care providers, 5 gynecologists, 4 breast radiologists, and 2 genetic counselors. Providers were predominantly female (100%), White (76%), and non-Hispanic (94%). Most providers (71%) had previously used breast cancer risk assessment models, and more than half (59%) had ordered screening breast MRI for women at high-risk for breast cancer. Among patients interviewed, 40% identified as Hispanic, 40% as non-Hispanic White, and 10% as non-Hispanic Black; 55% had previously undergone screening breast MRI.

Through qualitative analysis of semi-structured interviews with patients and providers, we identified three major themes related to screening breast MRI use: (1) Diffused responsibility impedes MRI screening initiation and knowledge; (2) Compounded barriers exist at multiple steps of the screening process for both patients and providers; and (3) Need for consolidated screening guidelines and accessible education.

### Theme 1

Diffused responsibility impedes MRI screening initiation and knowledge

### Subtheme 1: Variable use of breast cancer risk assessment tools

Among the 12 providers (71%) who had ever used a breast cancer risk calculator, only 8 reported using such tools regularly for women at elevated risk or with a family history of breast cancer. The most used models included Tyrer-Cuzick (n = 9), the Gail breast cancer risk assessment tool (n = 5), and Breast Cancer Surveillance Consortium (BCSC) risk calculator (n = 2). Seven providers (41%) explicitly cited time as a deterrent for using risk calculators.

I find it time consuming so it's not something I can put into my daily practice and if I have any patients who are high risk… then often my first step is actually just to refer them to the high risk clinic.

-PCP #1

I can't fill out Tyrer Cuzick with a patient. That would not be a good use of my time, and I don't have someone in my office who can do that.

-Radiologist #1

Many providers also expressed uncertainty in identifying and managing women at elevated risk, including limited familiarity with formal risk assessment tools. PCPs (83%, n = 5) and gynecologists (40%, n = 2) described discomfort with risk calculator use, infrequent utilization in practice, and reliance on referral to specialty clinics.

I think for some of these risk calculators, I think more education is needed. There's the one you mentioned I've never heard of.

-PCP #2

But in patients who tell me they don't have a family history, my understanding is it's still recommended to estimate their lifetime risk based on some of those other factors, and that's the gap that I feel like I'm missing out with my patients.

-PCP #3

Despite knowledge and familiarity with the risk assessment tools, no radiologists reported formal risk calculator use. They did express the need for more standardized approaches to data collection and risk assessment.

I can't fill out Tyrer Cuzick with a patient. That would not be a good use of my time, and I don't have someone in my office who can do that.

-Radiologist #1

We need a uniform way to collect data about risk and then assess risk and then act on it, but we do not have those things now.

-Radiologist #1

Conversely, one gynecologist described relying solely on risk estimates reported in mammography reports rather than independently calculating risk.

### Subtheme 2: Lack of ownership and reliance on breast health specialists

Most providers (82%, n = 14) indicated that while they may identify patients at risk, initiate breast cancer screening, or facilitate referrals to high-risk clinics, the primary responsibility for managing high-risk screening pathways should reside with another specialty or a dedicated breast health specialist (*e.g*., breast surgeon, medical oncologist, cancer genetic counselor). Most radiologists (75%, n = 3) stated that the referring clinicians (*e.g*., gynecologists or PCPs) should guide decision-making regarding supplemental breast imaging for high-risk patients, while also acknowledging that these providers are already stretched thin. In contrast, three gynecologists and two PCPs (45%) described their role as that of a gatekeeper, identifying patients who may require referrals to breast health specialists for supplemental screening. Two providers also believed radiologists should notify referring clinicians when screening breast MRI is indicated.

I feel like the primary care doctor's role, being more the referral to the specialist with expertise.

-PCP #3

I do feel like it's breast radiology's discretion.… If someone got a screening mammogram and an MRI was warranted or would be deemed helpful, I would hope that breast radiology would write that in a report and notify me…it's their expertise, not mine.

-PCP #2

Notably, some providers (35%, n = 6) expressed reluctance to assume responsibility for initiating MRI screening due to uncertainty in managing screening results and the absence of a clearly defined pathway.

We're ordering these tests. None of us are interpreting the test. We are basically functioning as technicians… just to coordinate care, rather than being an actual participant in subsequent breast testing and diagnosis. I can't be responsible for managing that level of risk and I shouldn't.

-GYN #1

One thing I would need is a clear pathway of what would happen if results came back high risk or positive….knowing that if someone is being told based on their breast MRI that they’re high risk…they would be able to get in and have a reasonably timed follow up appointment with a specialist.

-PCP #3

Everybody agrees we should be doing risk-based screening…some say we should do that ourselves. I do not want to do that myself when I then cannot offer the patient the next solution.

-Radiologist #2

### Subtheme 3: Contact with high-risk team makes process easier

The majority of patients interviewed (70%, n = 14) reported seeing a breast health specialist. Half (n = 10) indicated that these consultations improved their understanding of risk and increased confidence in decision-making.

I honestly feel like having a more tailored understanding of my own risk helps me to make that decision…otherwise it just feels kind of abstract…whereas speaking with a doctor…about me and my risk factors makes it feel much more like, you're telling me specifically what the next steps would be. So, I'm happy to kind of follow through with those

-Patient #10

Having the breast MRIs, that was the right choice for me. And I'm thankful that I do have a great oncologist who did take the time to sit with me and explain all these various preventive measures and help me determine what was right for me

-Patient #5

Providers similarly reported reliance on breast health specialists, with the majority (59%, n = 10) citing referral to a high-risk specialist as their primary resources for supporting risk-based screening and management of high-risk women. This included 83% (n = 5) of PCPs, and 60% (n = 3) of gynecologists. Three providers specifically cited discomfort with managing patients and potentially diagnosing conditions out of their scope as a primary reason for referral of high-risk patients.

Since I am a primary care doctor, I don't have any specialist or specific training in the area. I would be more confident in referring to a high-risk breast clinic to breast oncology or for genetics or for imaging.

-PCP #3

Usually once you need an MRI, I send you to a breast surgeon. You're too high risk for me. I'm prevention, like I'm not supposed to be diagnosing.

-GYN #1

### Theme 2

Compounded barriers exist at multiple steps of the screening process for both patients and providers

### Subtheme 1: Providers are limited by time and knowledge

Most PCPs and gynecologists reported knowledge gaps that contributed to hesitancy in ordering breast MRI screening. Among the 11 PCPs and gynecologists interviewed, 8 (72%) felt they lacked sufficient information to counsel patients effectively, particularly regarding MRI risks and follow-up questions. Time limitations with patients were also frequently cited, with 7 providers (41%) citing insufficient time to discuss screening as a structural constraint. Three mentioned that providers not having clear information makes patients more anxious and less likely to follow through with screening recommendations.

Patients may have more detailed questions that are not part of the training for general primary care. Some of them I may be able to answer, but some I may not. I always think it's better if you're going to do a screening, you need to have a plan in place for how the questions are going to be answered

-PCP #3

And that makes it difficult at the patient-provider level, because you're trying to explain all these different guidelines and telling the patient, “We can do shared decision making,” but the patient's like, “But I don't know what to do. I'm a patient, not a doctor.” So, it's challenging.

-PCP #4

### Subtheme 2: Uncertainty around insurance coverage for both the patient and provider

Health insurance coverage for screening breast MRI was also mentioned as a barrier by 40% (n = 8) of patients and 41% (n = 7) of providers. Providers were reluctant to order MRI screening due to concerns about cost and lack of clarity around insurance coverage.

I would say the biggest kind of obstruction in all of this in my experience has been the health insurance company.

-Patient #1

Doing testing that may have false positive rates or incur a lot of costs for my patient is a huge concern.

-PCP #2

### Subtheme 3: Emotional, procedural and logistical burdens on patients

Patients described several deterrents to MRI screening, including anxiety (40%, n = 8), physical discomfort (25%, n = 5), and scheduling or access challenges (30%, n = 6). Most physical complaints involved claustrophobia. Patient also described anxiety regarding potential false-positive findings and uncertainty about what to expect during the process. A small number of patients also expressed misconceptions about MRI screening, including fears of dying if jewelry was not removed before the exam (n = 2) and the belief that MRI increases radiation exposure (n = 1).

The ability to schedule it with, you know, like I know there's only so many MRI machines and you know, I just think that's hard to get in for a time.

-Patient #6

I was scared that it [MRI] would find something that the mammograms had always not found.

-Patient #8

### Theme 3

Need for consolidated screening guidelines and accessible education

### Subtheme 1: Willingness to order is limited by uncertainty

Although most providers recognized the benefits of screening breast MRI for high-risk women, variability in professional guidelines and persistent uncertainty around risk stratification limit implementation. All PCPs and two gynecologists reported that the guidelines were unclear, whereas all radiologists found them to be clear.

I think one additional challenge is that the field is constantly changing and I'm not able to stay up to date in terms of new risk like high-risk genetic syndromes or what the latest guidelines may be for screening a high-risk population

-PCP #1

Providers referenced multiple guidelines sources (*e.g*., American College of Obstetrics and Gynecology [ACOG], American Cancer Society [ACS], American College of Radiology [ACR], National Comprehensive Cancer Network [NCCN], Society of Breast Imaging [SBI], and United States Preventive Services Task Force [USPSTF]). Notably, all PCPs indicated that additional education and access to concise reference tools would increase their confidence in ordering MRI screening.

So honestly like if there were guidelines that I could follow that will like tell me like what is the appropriate test to order and interval…it doesn't seem like it would be that like big of a lift so I would feel comfortable knowing that my patient is high risk, they require a little bit of extra, extra screening.

-PCP #5

I would be more likely to offer [MRI screening] if there was something in the EMR that clearly said this is the patient's lifetime risk. Would you like to order this additional testing? Yes. And then it automatically brings me to the order set and I'd order that…I would order it much more often

-GYN #2

### Subtheme 2: Integration into the electronic medical record (EMR) could improve uptake

A common barrier for providers was the lack of a unified system that integrates risk assessment tools, screening recommendations, and referral workflows. Most providers (71%, n = 12) reported that EMR-integrated tools would help streamline the breast MRI screening process. When asked about adopting new tools or technologies in their practice, 71% reported being very open, while 29% reported being somewhat open.

…an epic dot phrase would be the most helpful…something I could pull up quickly or maybe if it inputted with like a recommendation based on the risk calculation

-PCP #1

I feel like if it were something that were embedded in Epic, so that like when I'm working in the chart, I can automatically see the guidelines alongside of me being able to put in orders

-PCP #5

### Subtheme 3: Need for clearer education on MRI benefits, downstream consequences and financial considerations

Patients identified several factors influencing their decisions to undergo or consider breast MRI screening. About a third of patients (n = 7) were motivated by the perceived benefits of screening, while 20% (n = 4) expressed hesitation due to uncertainty about downstream consequences, such as false positive results or the need for additional testing. A few (n = 4) reported that their decision was based primarily on their physician’s recommendation. Patients also highlighted recurring knowledge gaps, including concerns about radiation exposure, interpretation of screening results, limitations of mammography for certain individuals, and expectations for the breast MRI screening process.

How much more does the MRI see than a digital mammogram?

-Patient #7

If mammograms have radiation, why not do 2 MRIs instead?

-Patient #6

Providers also emphasized the need for patient educational materials to better guide individuals through the screening process (35%, n = 7). Many reported being limited by time or gaps in their own knowledge and said it would be helpful to have clear, accessible resources to support patient counseling on screening topics.

…it's hard for patients to be proactive in this space, or for them to like attend to this part of their health if there's not like clear outreach on our part to guide them

-Radiologist #1

Although patient educational materials were identified as a clinical need, two providers emphasized that inconsistent messaging across clinicians can undermine patient trust in the healthcare system and hinder patients’ ability to actively participate in their screening choices, underscoring the need for provider education alongside patient-directed resources.

I think we need provider education, making sure that we're all on the same page with screening recommendations because providing conflicting recommendations to patients just decreases their trust in the medical system and ultimately leads to non-adherence and poor patient outcomes

-Radiologist #3

## Discussion

This pre-implementation study is among the first to systematically integrate patient and provider perspectives on breast MRI screening barriers to directly inform development of clinical decision support (CDS) tools. Although all participants were receptive to breast MRI screening for women at high risk for breast cancer, uptake was constrained by multilevel barriers including knowledge gaps, fragmented clinical ownership and structural challenges. These findings underscore the need for targeted interventions that address system-, provider- and patient-level barriers, while equipping PCPs and gynecologists with the CDS tools necessary to implement risk-based breast cancer screening in routine clinical practice.

### Provider-Level Barriers

Despite strong evidence that supplemental breast MRI screening detects earlier stage breast cancer and reduces interval cancers among high-risk women, uptake remains suboptimal [4, 5]. Our findings help explain this gap by identifying breakdowns at key steps of the screening pathway, particularly in risk stratification, clinical knowledge and care coordination. PCPs reported limited comfort navigating inconsistent guidelines, counseling patients on risks and benefits of MRI screening and managing abnormal results. PCPs and gynecologists positioned themselves as navigators of a complex system rather than initiators of risk assessment or managers of downstream screening decisions. This discomfort led to heavy reliance on referral to breast health specialists, with most PCPs and gynecologists interviewed deferring management to high-risk clinics.

Radiologists, in turn, consistently viewed risk stratification as the responsibility of the referring clinical team, positioning themselves as interpreters of imaging rather than initiators of risk-based screening. Their non-use of formal risk calculators reflects a scope-of-practice boundary rather than a knowledge deficit and, ultimately, the result is a misalignment in perceived responsibility. PCPs and gynecologists defer to specialists, while radiologists defer to referring clinicians, creating a gap in which no single provider group, besides breast health specialists, owns the initiation of breast MRI screening. This fragmentation in care is consistent with prior work demonstrating that MRI screening rates are substantially higher in high-risk clinics than in primary care practice and that only 12% of PCPs recommend MRI screening for eligible high-risk women [10].

Importantly, reliance on specialty clinics may not be scalable, particularly in non-academic and community settings. Although breast health specialists remain essential for patients with complex risk profiles, such as those with germline pathogenic variants in hereditary breast cancer predisposition genes, patients classified as high-risk based on Tyrer-Cuzick score or family history alone may not require specialist referral. Instead, these patients could be managed by a trained gynecologist or PCP equipped with adequate decision support, such as systematic breast cancer risk assessment incorporated into the EMR and providing clinicians with concise guidelines on how to manage breast cancer risk.

### Patient-Level Barriers

On the patient side, individuals faced barriers throughout the screening process, including emotional burden, procedural concerns, and logistical challenges. Anxiety about potential false positive results was common. Physical discomfort, particularly claustrophobia, and scheduling difficulties further deterred screening. Several patients also held misconceptions about MRI, including fears about radiation exposure and the rationale behind MRI screening, which they attributed to information learned from external sources rather than clinical counseling.

Many of these barriers reflect the limits of a single clinical encounter to convey complex, individualized risk information. Patients frequently described accepting their provider's screening recommendations at face value without fully understanding their personal risk or feeling prepared to ask informed questions. This finding aligns with prior research showing that limited health literacy and insufficient patient-provider communication contributes to the low uptake of supplemental cancer screening [22-24]. Notably for individuals interviewed in this study, consultation with a breast health specialist eased the uncertainty by providing patients with specialized knowledge and dedicated counseling time, resulting in greater clarity and confidence in their screening decisions. Together, these findings support the value of accessible patient educational materials that can reinforce provider counseling, correct common misconceptions, and prepare patients to engage more actively in conversations with their care team, particularly when specialist access is limited.

### Implications for Implementation

While prior work has broadly characterized barriers to breast cancer screening, this qualitative approach yielded the granular, patient- and provider-specific detail needed to directly inform intervention design. As depicted in Tables 3 and 4, each barrier identified in our interviews will be mapped to a targeted feature within *BNAV* or *RealRisks*, ensuring that the development of intervention components is grounded in the actual barriers described by our study population.

For providers, planned resources include quick-reference tip sheets summarizing risk-based breast cancer screening guidelines, EMR-integrated risk alerts, and automated pre-visit calculation of personalized breast cancer risk according to the Tyrer-Cuzick model via an EMR-integrated Ambry CARE^®^ platform. These features directly address the gaps in guideline familiarity and inconsistent use of risk assessment tools that emerged as themes in our interviews. By enabling risk assessment to occur before the clinical encounter in primary care or mammography screening visits, these tools may reduce the time burden that providers frequently cited as a deterrent. *BNAV* will also incorporate structured talking points and decision support content designed to build PCP and gynecologist confidence in managing high-risk patients directly, while clarifying when referral to a high-risk clinic remains warranted.

On the patient side, the *RealRisks* decision aid delivers tailored education and risk communication. Ongoing module development seeks to provide information on the available screening modalities, address common misconceptions, clarify what to expect with each breast imaging modality, and offer guidance around logistical barriers such as insurance coverage and scheduling. For example, one module will contextualize the mammographic radiation exposure by comparing it to that of a crosscountry flight, drawing on established risk communication principles to make information more tangible. Prior work has demonstrated that *RealRisks* improves accuracy of breast cancer risk perceptions without increasing patient anxiety, and prior usability studies have shown similar effectiveness across racially and ethnically diverse populations, suggesting that the platform is well suited to heterogenous populations[14, 25].

### Strengths and Limitations

Strengths of this study include the integration of quantitative survey data with qualitative insights from semi-structured interviews, capturing perspectives from a diverse patient population and a range of provider specialties involved in breast cancer screening. The direct mapping of findings to our intervention components represents a methodological strength that enhances the relevance of our findings. However, several limitations should be noted. The provider sample was entirely female, lacked demographic diversity and was drawn from a single academic medical center, which may limit generalizability to community based and rural practice settings where barriers to MRI screening may differ. Additionally, the small number of radiologists (n = 4) and genetic counselors (n = 2) precludes within-specialty comparisons. Over half of patients interviewed had previously undergone breast MRI screening (55%), and over half of providers had ordered it (59%). As a result, our sample was skewed toward already engaged individuals and barriers among less-engaged individuals may be underrepresented.

## Conclusions

In summary, these results identify key multilevel barriers to breast MRI screening including fragmented clinical ownership, provider knowledge gaps and patient misconceptions and logistical challenges, which underscores the need for interventions that integrate personalized risk assessment, CDS tools, and patient education into routine care. Addressing these gaps is essential to implementing risk-based breast cancer screening and improving early detection among high-risk women. Future work will involve iterative design and usability testing of the *RealRisks* screening module, which will be available in English and Spanish and tailored to patients with varying health literacy and educational backgrounds. Following usability testing, we will conduct a randomized controlled trial to evaluate the effectiveness of these modules in improving patient outcomes and screening-related decision-making.

## Figures and Tables

**Table 1:** **Provider Characteristics** (N = 17)

**Mean Age, years (SD)**	**42.6 (12.7)**
Sex, N (%)	
Female	17 (100)
Ethnicity, N (%)	
Hispanic/Latina	1 (6)
Non-Hispanic	16 (94)
Race, N (%)	
Black/African American	3 (18)
White	13 (76)
Other	1 (6)
Medical Specialty, N (%)	
Primary Care Provider	6 (35)
Gynecologist	5 (29)
Breast Radiologist	4 (24)
Cancer Genetic Counselor	2 (12)
Previously Used Breast Cancer Risk Assessment Models, N (%)	
Yes	12 (71)
No	5 (29)
Ordered Breast MRI, N (%)	
Yes	10 (59)
No	7 (41)

**Table 2:** **Patient Characteristics** (N = 20)

**Mean Age, years (SD)**	**51.8 (14.8)**
Ethnicity, N (%)	
Hispanic/Latina	8 (40)
Non-Hispanic	12 (60)
Race, N (%)	
Asian	1 (5)
Black/African American	5 (25)
White	11 (55)
Other	3 (15)
Primary Language, N (%)	
English	16 (80)
Spanish	4 (20)
Family History of Breast Cancer, N (%)	
Yes	19 (95)
No	1 (5)
Prior Consultation with Breast Health Specialist or Genetic Counselor, N (%) Yes	14 (70)
No	5 (25)
Unsure	1 (5)

**Table 3:** Provider Barriers to Breast MRI Screening and Intervention Components

Clinical Workflow Step	Provider Barriers to MRI Screening	Proposed Interventions
Breast cancer risk assessment	Inconsistent use of risk assessment tools	Implement Ambry CARE^®^ prior to primary care and mammography visits to calculate Tyrer-Cuzick score
Cancer screening guideline interpretation and ordering	Guideline unfamiliarity and inconsistency across professional societies	Create “Tip Sheets” for providers as a quick reference for risk-based breast cancer screening practices
Counseling on breast cancer risk management	Clinical burden and time constraints during clinical encounter	Establish Epic smartphrases that incorporates screening recommendations based upon personalized Tyrer-Cuzick score
Ordering screening tests	Diffusion of provider responsibility	Include a personalized Tyrer-Cuzick scores in mammogram reports with clear next-step guidelines
Result management	Provider discomfort counseling patients on being high-risk and recommendations for enhanced breast cancer screening	Talking points/FAQ module in *BNAV*
Referrals	Overreliance on specialist referral due to unclear pathway following abnormal or high-risk results	*BNAV* decision support tool to build PCP/gynecologist comfort and knowledge in managing high-risk patients directly; clear indications for referral to a high-risk clinic

**Table 4:** Patient Barriers to Breast MRI Screening and Intervention Components

Patient Barriers to MRI Screening	Proposed Intervention in *RealRisks* Screening Module
Uncertainty about various screening modalities	Side-by-side visual comparison of screening modalities; emphasis on the added sensitivity of breast MRI in high-risk populations
Procedural anxiety and physical discomfort	Module walking patients through preparation, positioning, and what to expect during the screening
Fear of false positives and uncertainty about interpreting abnormal results	Module explaining false positive using an accessible analogy (*e.g*., fire alarm) with a plain-language overview of the BIRADS scoring system
Concerns about insurance coverage and out-of-pocket costs	Actionable guidance on how to verify coverage, navigate prior authorization and identify financial assistance resources

## Abbreviations

ACR: American College of Radiology
ACS: American Cancer Society
BCSC: Breast Cancer Surveillance Consortium
EHR: Electronic health record
GC: Genetics counselor
GYN: Gynecologist
MRI: Magnetic resonance imaging
PCP: Primary care provider
USPSTF: US Preventative Services Task Force
